# Assessing the impact of misinformation during the spread of infectious diseases

**DOI:** 10.1038/s41598-025-18457-1

**Published:** 2025-10-06

**Authors:** Alejandro Bernardin, Tomas Perez-Acle

**Affiliations:** 1 Computational Biology Lab (DLab), Fundación Ciencia & Vida, Universidad San Sebastián, Av. Del Valle Norte 725, 8580702 Santiago, Chile; 2 Facultad de Ingeniería, Universidad San Sebastián, Av. Del Condor 720, 8580704 Santiago, Chile

**Keywords:** Infectious disease, Epidemic, Misinformation, Ebola, Diseases, Health care, Mathematics and computing

## Abstract

In today’s digital era, the internet offers unprecedented access to information, but it also accelerates the spread of misinformation. Nowhere is this more problematic than in public health, as the COVID-19 pandemic clearly demonstrated. Misinformation can erode trust in science and health authorities, leading people to disregard expert guidance and adopt unverified treatments that endanger population health. We examine how misinformation alters the course of an infectious disease outbreak by modeling the simultaneous uptake of preventive measures and engagement in harmful behaviors. The model captures the competing influences of accurate information and misinformation on individual decision making. Our results show that even a modest influx of misinformation can greatly amplify disease transmission, deepening the epidemic’s severity. These findings highlight the urgent need for robust strategies to curb misinformation and support public health interventions during health crises.

## Introduction

The exponential growth of the internet has dramatically expanded channels for disseminating information, amplifying both reliable reporting and false or misleading content^1,2^. Here, we define *factual information* as evidence-consistent guidance and *misinformation* as an umbrella term for claims that contradict the best available evidence at the time of sharing, irrespective of intent^2^. The stakes of spreading misinformation become particularly high in public health contexts, where access to reliable information is paramount, especially concerning infectious disease outbreaks. This scenario unfolds within a complex web of dynamics heavily influenced by human behavior, which in turn is shaped by the information that individuals receive, primarily through mass media, the internet, and social networks^3^.

The COVID-19 pandemic has given rise to “infodemics”, as defined by the World Health Organization: an overwhelming mix of accurate and misleading information flooding digital platforms and social media^4,5^. This phenomenon illustrates the dual impact of digital information on public health crisis management. Specifically, the spread of misinformation on COVID-19, including baseless treatments, speculative ideas, and conspiracy theories^6–8^, highlights the substantial public health risks posed by misinformation. Therefore, the role of the internet and social networks in distributing information presents a paradox, as they have the power to either dampen or intensify the spread of infectious diseases^9^.

To elucidate the impact of misinformation during health crises, such as infectious disease outbreaks, our research examines the dynamics of disease transmission among human populations. We emphasize the dual role of factual information and misinformation, which can respectively foster preventive or harmful behaviors. Our approach involves a SEIRD compartmental model embedded in an Agent-Based Modeling (ABM) framework, featuring mechanisms for information decay and differentiating between factual information and misinformation. Furthermore, it incorporates behavioral responses, classified as either preventative or harmful. Through this framework, our objective is to analyze the impact of disseminating both factual information and misinformation on the spread of an infectious disease.

We have chosen Ebola Virus Disease (EVD) as the focus of our case study. EVD is characterized by a high mortality rate and poses a significant challenge to public health. To support our selection in the COVID-19 era, it is crucial to consider that the basic reproductive number ($R_0$) of EVD is below 1.5^10^, meaning that each infected person transmits the disease to, on average, 1.5 other individuals. This suggests that with effective interventions to reduce $R_0$ close to 1 (stop the spread of the infection), containment of EVD could be feasible. The selection of EVD is emphasized by its unique ability to trigger profound social and behavioral reactions—such as panic, discrimination against healthcare professionals, excessively stringent public policies, and widespread skepticism toward scientific findings—amplified by media and political rhetoric^11,12^. This complexity makes EVD an exemplary case for studying the interplay between infectious disease dynamics and societal response to misinformation dissemination.

The EVD outbreaks between 2014 and 2016 highlighted the essential role of public health strategies in the management of the disease, especially given the lack of a universally effective vaccine or treatment for most EVD strains when those outbreaks occurred^13^. This period demonstrated the intricate relationship among disease transmission, the spread of misinformation, and societal behaviors—a focal point of our research. The prevalence of misinformation and the consequent erosion of trust in institutions during these outbreaks had a profound impact on public reactions and the success of health policies. This underscores the importance of analyzing the interaction between the dispersion of factual information and misinformation within our model^14,15^.

Given the progression since the last major outbreaks, it is noteworthy that while treatments and vaccines for EVD have since advanced, the disease remains a global health concern^16,17^. This backdrop, together with the unique set of characteristics of EVD, such as $R_0$, makes it an ideal subject to study the impacts of factual information, misinformation, and behavioral responses on disease containment and public health strategy.

Our findings suggest that misinformation can significantly alter the course of an infectious disease’s spread, amplifying its impact on populations even with minimal exposure. This phenomenon adds an extra, challenging layer of complexity to the already formidable task of containing epidemics.

## Methods

### Model overview

Compartmental models, fundamentally established by the seminal contributions of Kermack and McKendrick in 1927^18^, are the workhorse of theoretical epidemiology. Individuals are grouped as *susceptible* (S), *infected* (I), and *removed* (R) based on their health status over time. The *removed* category comprises individuals who no longer participate in the disease dynamics due to recovery, acquired immunity, or death. Adaptations of these models, including but not limited to SIS (susceptible–infected–susceptible), SEIRD (susceptible–exposed–infected–removed–death), and SEIRHVD (susceptible–exposed–infected–removed–hospitalized–mechanically_ventilated–death), are designed to accommodate the progression stages of various diseases^19–21^. A critical component of these models is the *infection rate*, $\beta$, which quantifies the transition of individuals from susceptible to exposed or infected categories, depending on the disease. Although $\beta$ is often assumed to be constant for simplification, this assumption is primarily for analytical tractability. In practical scenarios, $\beta$ is subject to temporal variations influenced by interventions, changes in pathogen virulence, and the development of herd immunity^22,23^. Recent literature suggests that $\beta$ not only depends on biological factors but also on the quality and quantity of information individuals have about the infection at any given time^24–27^. To explore this, we introduce two parameters to independently account for the influence of factual information (*x*(*t*)) and misinformation (*y*(*t*)) on disease dynamics, where *x* and *y* range from 0 (no information/misinformation) to just below 1 (fully informed/misinformed).

### Behavioral-information factor (BIF)

Following our previous approach^28^, yet extending it to include misinformation, we propose $\beta$ as follows:

1 $$\begin{aligned} \beta \rightarrow \beta \Lambda (x,y), \end{aligned}$$

where $\Lambda$ is the *behavioral-information factor, BIF*, a function of time through *x*(*t*) and *y*(*t*). Although the exact form of $\Lambda$ is not specified, it should meet certain criteria: (1) $\Lambda (x,y)\ge 0$, as $\beta$ is always positive; (2) $\Lambda (x,y)$ approaches 1 as both *x* and *y* approach 0, reflecting the baseline infection rate; (3) $\Lambda$ is analytic at (0, 0), allowing expansion as a Taylor series; and (4) the effects of information and misinformation on $\Lambda$ are inversely related, modeled as

2 $$\begin{aligned} \Lambda (x,y) = \Lambda (0,0) + \partial _x \Lambda (0,0)x + \partial _y \Lambda (0,0)y + H.O.T., \end{aligned}$$

where *H*.*O*.*T*. denotes *higher order terms* of the Taylor series. We can visualize $\Lambda (x,y)$ in Supplementary Figure S6.

### Information-decay kinetics

To elucidate the dynamics of factual information (*x*) and misinformation (*y*) over time, we define *x*(*t*) and *y*(*t*), as $\rho _a^{i(t)}$ and $\rho _u^{j(t)}$, namely the *awareness decay constant* and the *misinformation decay constant*, respectively. Here, *i* and *j* represent the *quality* of factual information and misinformation, respectively, which decays over time or as it is passed from one individual to another. This decay is modeled by $X_i+X_{k>(i+1)} \rightarrow X_i+X_{i+1}$ as proposed in^29^, illustrating that when an individual with lower-quality information (*k*) interacts with someone possessing higher-quality information (*i*), the resultant information is of quality $i + 1$, slightly diminished from the original (larger numbers imply lower quality). Notably, high-quality information ($i = 0$) is generated in newly infected individuals, emphasizing the role of direct experience in fostering high-quality awareness about the epidemic^30^. We emphasize that by the “quality” of factual information or misinformation, we refer to its fidelity to the original source. In this context, information that originated long ago and has been transmitted through multiple intermediaries tends to lose quality, that is, it may no longer contain all the original details or may have lost part of its content. Consequently, high-quality information is that which closely preserves the content and accuracy of the original source, regardless of whether it is factual information or misinformation.

Importantly, our model conceptualizes awareness as knowledge about the epidemic that motivates preventive actions, a critical factor in disease control^31^. Consistent with the general definition of misinformation given above, we *operationalize* (in the public-health context) misinformation as evidence inconsistent beliefs that motivate harmful (risk-promoting) behavior, thereby undermining control efforts. This is an operational refinement, not a second definition, restricting “misinformation” to the behaviorally relevant subset used in the model.

Now, simplifying the BIF ($\Lambda$) to its first-order approximation in *x* and *y*, since both variables remain below 1 for all *t*, and defining $\gamma _a = -\partial _x \Lambda (0,0)\ge 0$ and $\gamma _u = \partial _y \Lambda (0,0)\ge 0$, we can reformulate the adjusted infection rate ($\beta$) as

3 $$\begin{aligned} \beta \rightarrow \beta \left( 1-\gamma _a\rho _a^i+\gamma _u\rho _u^j\right) . \end{aligned}$$

This formulation allows us to consider the original model by Funk et al.^29^, where $\beta \rightarrow \beta \left( 1-\rho _a^i\right)$, as a special case within our broader framework, achieved by setting $\gamma _a=1$ and $\gamma _u=0$. This extension incorporates the impacts of both factual information and misinformation on disease spread, enriching the model’s capacity to explore epidemic dynamics. We summarize all the parameters of our model in Table 1.

**Table 1:** Summary of model parameters used in the ABM–SEIRD framework. We present the parameters for the infectious disease and communication models. The dimensionless parameters are indicated with a “–”. More details in Supplementary Material S2.

Parameter	Value	Units	Description
$T_E$	11	days	Incubation period (E $\rightarrow$ I)
$T_I$	6	days	Infection period (I $\rightarrow${R, D})
$T_D$	4	days	Death-to-burial period (D $\rightarrow$ burial)
$f$	0.7	–	Fatality rate (I $\rightarrow$ D)
$\beta _I$	0.25	$\hbox {day}^{-1}$	Infection rate (I $\rightarrow$ S)
$\beta _D$	0.20	$\hbox {day}^{-1}$	Infection rate from corpses (D $\rightarrow$ S)
$r_{ai}$	[0,1]	–	Awareness–information state
$r_{ui}$	[0,$\infty$)	–	Unawareness–information state
$\rho _a$	[0,1]	–	Awareness decay constant
$\rho _u$	[0,1]	–	Misinformation decay constant
$q_{ai}$	[0,$\infty$)	–	Awareness–information quality constant
$q_{ui}$	[0,$\infty$)	–	Unawareness–information quality constant
$\gamma _a$	[0,1]	–	Preventive behavior coefficient
$\gamma _u$	[0,$\infty$)	–	Harmful behavior coefficient
$\alpha _a$	[0,1]	–	Ratio of informed individuals with factual information
$\alpha _u$	[0,1]	–	Ratio of informed individuals with misinformation

The transformation of information into actionable behavior is a pivotal aspect of our model. Information, irrespective of its persistence, indicated by the awareness decay constant $\rho _a$ for factual information and misinformation decay constant $\rho _u$ for misinformation, must ultimately influence behavior. This underscores a fundamental principle: *the possession of information alone is insufficient unless it translates into behavioral change*^32,33^. In our framework, $\gamma _a$ and $\gamma _u$ serve as metrics quantifying preventive and harmful behaviors, respectively. This differentiation is crucial as it acknowledges the varying effectiveness of behaviors in combating or exacerbating disease spread.

For instance, preventive behaviors vary in efficacy, from basic hygiene practices to vaccination, necessitating a model that is flexible enough to simulate different strategic interventions. Conversely, harmful behaviors also exhibit a spectrum of impact. Notably, behaviors such as avoiding vaccination can significantly affect disease dynamics. More extreme examples, such as “measles parties” discussed in the literature^34,35^, highlight scenarios where misinformation leads to intentionally risky behavior, aiming at natural immunity through infection. These examples highlight the potential of our model to study the effects of various types of behaviors, both preventive and harmful, on the spread of disease. It’s important to highlight that the harmful behavior coefficient, denoted by $\gamma _u$, can potentially reach infinite values. This reflects the reality that harmful behaviors can exacerbate the infection rate exponentially, unlike protective behaviors $\gamma _a$, which, by their nature, cannot reduce the infection rate to negative values. This distinction underscores the potentially boundless impact of detrimental actions on the spread of an infection.

Crucially, our model distinguishes between the decay constants of factual information $\rho _a$ versus misinformation $\rho _u$. Empirical evidence suggests that misinformation, often more sensational and emotionally charged, tends to spread more widely and persist longer than factual information^1,36^. As an example, we have the “salt-water” cure that urged people to bathe and drink hot salt water because ’Ebola is now airborne’, leading to reported deaths and hospitalizations, an archetypal high arousal, urgent frame^37^. As we see, this distinction is not merely academic, but reflects observed phenomena, which warrants a different treatment of $\rho _u$ and $\rho _a$ in our model. Because of this, we assume $\rho _u > \rho _a$, indicating that misinformation has a more prolonged influence compared to factual information. This definition will become important for establishing a more realistic approach when analyzing the outcomes of our simulations (see below).

Extensive research has been conducted on the spread of EVD, involving both the collection of empirical data and the development of mathematical models to understand its temporal dynamics^12,38–45^. The SEIRD model, which accounts for the significant roles of both the incubation period and the infectivity of deceased individuals, has been particularly effective in capturing the disease’s behavior. This model’s parameters were initially derived from data that did not consider the impact of information flow on disease transmission^40^. In response to this gap, our study employs an ABM framework designed to simulate the spread of EVD in a population while incorporating the competing dynamics of factual information and misinformation. This approach enables a more nuanced understanding of how factual information and misinformation can influence disease spread. Specifically, we extend the model to adjust the infection rates from infected to susceptible individuals ($\beta _I$) and from deceased to susceptible individuals ($\beta _D$), incorporating the effects of information and misinformation as follows:

4 $$\begin{aligned} \beta _I\rightarrow \beta _I(1 - \gamma _a \rho _a^i + \gamma _u \rho _u^j), \quad \beta _D\rightarrow \beta _D(1 - \gamma _a \rho _a^i + \gamma _u \rho _u^j), \end{aligned}$$

where $\gamma _a$ and $\gamma _u$ are BIF parameters that quantify the effects of preventive and harmful behaviors, respectively, across both live and deceased individuals. Similarly, $\rho _a$ and $\rho _u$ are BIF parameters that quantify the decay of factual information and the decay of misinformation, respectively. The uniform application of BIF underscores our model’s approach to studying disease transmission, emphasizing the critical role of information dynamics in public health responses to epidemics.

### Implementing the model in an ABM framework

Based on the ABM capabilities of NetLogo 6.1.1^46^, a platform renowned for its versatility in multi-agent simulations and robust support community, we designed a simulation encompassing 10,000 agents. NetLogo’s support for spatially embedded agent dynamics is particularly advantageous for epidemiological simulations, where the spatial proximity between agents significantly influences disease spread^47–49^.

In our model, each agent is defined by four primary parameters: spatial position $z_i$, epidemiological state $Q_i$, awareness-information state $r_{ai}$, and unawareness-information state $r_{ui}$ (representing the possession of misinformation that promotes harmful, risk-increasing behavior), with *i* indicating the individual agent. The epidemiological states are derived from the SEIRD model, accommodating susceptible *S*, exposed *E*, infected *I*, recovered *R*, and deceased states *D*. The awareness-information and unawareness-information states quantify the factual information and misinformation an agent has about EVD, respectively, influencing both their behavior and susceptibility.

Agents perform a random walk on a 2D torus (periodic boundaries). This approach ensures that, over time, agents disperse uniformly across the space, albeit theoretical convergence to a uniform distribution is only achieved asymptotically. While agents primarily move independently, their epidemiological and information states evolve based on interactions, simulating the transmission of both disease and information. Minor modifications from our prior work^28^ include adjustments to the rate of information decay and the introduction of more nuanced agent behaviors based on their information state, enhancing the model’s realism and applicability to real-world scenarios. Although our simulations extend to 1000 days, this period is selected to balance computational feasibility with the need to observe the long-term dynamics of EVD spread and information flow, acknowledging that this timeframe may offers merely a snapshot rather than a comprehensive endpoint of epidemic progression. Each set of parameters of the simulation was run 100 times, to then calculate a mean and a standard deviation.

At the outset of the simulation, agents are uniformly distributed across the 2-torus space, which is subdivided into $85\times 85$ square patches, each with a side length of 1. This spatial arrangement creates a grid-like environment conducive to simulating disease transmission and information dissemination, with interactions restricted to agents sharing the same patch. The choice of this patch size and number was strategically made to emulate interaction rates that align with the SEIRD model outcomes based on ordinary differential equations (ODEs) detailed by Weitz and Dushoff^40^, specifically tailored for understanding the EVD dynamics during the 2014–2016 outbreak. For details on the parameters used, see S1.

In terms of epidemiological dynamics, our simulation delineates two primary processes: infection dynamics and state transition dynamics. As mentioned before, the agents may be in one of five epidemiological states: $Q_i\in \{S,E,I,R,D\}$. The infection dynamics is triggered when susceptible agents encounter infected or deceased agents within the same patch. This event initiates a probabilistic transition, governed by a Monte Carlo algorithm, from the (*S*) to the (*E*) state, denoted as $S\rightarrow E$. Specifically, during these encounters, we generate a random number $\nu$ from a uniform distribution ${\mathcal {U}}(0,1)$ for each *S* agent present. This number is then compared against the infection probability $\beta _{I,D}(1-r_{ai}+r_{ui})$. If $\nu <\beta _{I,D}(1-r_{ai}+r_{ui})$, the transition to the *E* state occurs.

The infection probabilities $\beta _I$ and $\beta _D$, for $S\rightarrow E$ transitions due to (*I*) and (*D*) agents, respectively, are adapted from the parameters identified by Weitz and Dushoff^40^ for the EVD outbreak. These parameters incorporate the impact of awareness-information

$$\begin{aligned} r_{ai}(t)=\gamma _a\rho _{ai}^{q_{ai}(t)} \end{aligned}$$

and unawareness-information

$$\begin{aligned} r_{ui}(t)=\gamma _u\rho _{ui}^{q_{ui}(t)} \end{aligned}$$

states on the infection process, thereby integrating the dynamics of information flow with epidemiological spread. Given the simulation’s setup, interactions are predominantly pairwise, owing to an average agent density per patch of approximately 1.38, a condition that also holds true for information exchange dynamics.

Apart from the susceptible state, agents experience transitions such as $E\rightarrow I$, $I\rightarrow R$, $I\rightarrow D$, and $D\rightarrow \emptyset$, reflecting the progression from exposure to infection, recovery or death, and eventually removal from the simulation (signifying burial). Transitions from *E* to *I* and from *D* to $\emptyset$ occur deterministically after periods $T_E$ and $T_D$, respectively. Conversely, the transitions $I\rightarrow R$ and $I\rightarrow D$ happen after $T_I$ days, determined probabilistically at rates $1-f$ and *f*, respectively, illustrating the uncertainty in an infected individual’s outcome. Furthermore, the dynamics of these transitions are influenced by each agent’s awareness-information state $r_{ai}\in [0,1]$, and unawareness-information state, $r_{ui}\in [0,\inf )$. These states dynamically affect the transition probabilities by altering the rates $\beta _I$ and $\beta _D$. The temporal evolution of an agent’s awareness-information and unawareness-information states, represented by $r_{ai}(t) = \gamma _a\rho _{ai}^{q_{ai}(t)}$ and $r_{ui}(t) = \gamma _u\rho _{ui}^{q_{ui}(t)}$, respectively, models how the quality of information—whether factual information or misinformation—waxes or wanes over time. This variation in information quality and its impact on the infection rates $\beta _{I,D}$ highlight the intricate relationship between the spread of information and the dynamics of the epidemic. Additionally, $q_{ai}(t)\in {\mathbb {N}}$ denotes the *awareness-information quality constant* for agent *i*, a time-varying measure with $q_{ai}(t)=0$ indicating the peak quality of awareness-information, a concept paralleled in the *unawareness-information quality constant*, $q_{ui}(t)$.

To account for heterogeneity in agent responses, the model allows that each agent’s awareness decay constant $\rho _{ai}$ and misinformation decay constant $\rho _{ui}$ are sampled from distributions $p(\rho _a)$ and $p(\rho _u)$, where in this case *p*(*x*) is a delta distribution, for simplicity. These distributions aim to reflect societal characteristics, suggesting that a society’s trust levels, in its institutions, media, and amongst individuals can significantly impact the collective behavior in response to an epidemic. Although a direct method to quantify these sociocultural factors into specific distributions for $\rho _{a,u}$ remains elusive, the model posits that societies with higher trust are likely to maintain higher levels of awareness longer than those with lower trust levels, especially when faced with crucial information on how to handle disease spread^50^. This detailed framework for agent transitions and the integration of awareness and unawareness dynamics into the model not only enhances the realism of simulated epidemic scenarios but also opens avenues for exploring the effects of societal traits on epidemic outcomes.

In our model, agents acquire new factual information and misinformation through three principal mechanisms: (1) exposure to a global source that periodically disseminates factual information or misinformation, thereby resetting the quality constant $q_{(a,u)i}(t)$ to zero for all recipients. This reset reflects the reception of an authoritative, lossless broadcast (e.g., an official Ministry of Health briefing), which completely replaces any prior uncertainty, rather than merely incrementally improving the existing information. (2) interaction with other agents within the same patch, where factual information or misinformation of superior quality is transferred, leading to $q_{(a,u)i}(t)=q_{(a,u)j}(t)+1$; and (3) upon acquiring the disease, where exposed agents automatically receive high-quality information, reflected by $q_{ai}(t)=0$. Factual information and misinformation quality degrade over time, with quality decreasing by one unit per simulation time step. Accordingly, the evolution of an agent’s information quality over time is described by:

5 $$\begin{aligned} q_{ai}(t+1) ={\left\{ \begin{array}{ll} 0 & \text {if agent }i\text { receives global factual information or is newly infected,}\\ q_{aj}(t)+1 & \text {if agents }i\text { and }j\text { interact and }q_j(t)<q_i(t),\\ q_{ai}(t)+1 & \text {otherwise.} \end{array}\right. } \end{aligned}$$

Similarly, the dynamics of misinformation follow a parallel pattern, albeit with nuanced differences to reflect the distinct nature and spread of misinformation:

6 $$\begin{aligned} q_{ui}(t+1) ={\left\{ \begin{array}{ll} 0 & \text {if agent }i\text { is targeted by global misinformation,}\\ q_{uj}(t)+1 & \text {if agents }i\text { and }j\text { interact and }q_j(t)<q_i(t),\\ q_{ui}(t)+1 & \text {otherwise.} \end{array}\right. } \end{aligned}$$

This model posits that factual information and misinformation are transferred from agents possessing higher-quality content to those with lower-quality content during interactions, underpinning the model’s communication dynamics. This assumption is consistent with the two-step flow: more informed opinion leaders, those with greater media exposure and knowledge (high-quality), tend to transmit information to less active followers (low-quality)^51,52^. Similarly, classical rumor-spreading models^53^ formalize this directional flow, where an informed “spreader” converts an “ignorant” individual upon contact. Crucially, in our model, this dynamic can operate both through factual information and through misinformation. Importantly, these exchanges are based on the condition that both participating agents are alive: $\{S, E, I, R\}$.

**Fig. 1:**
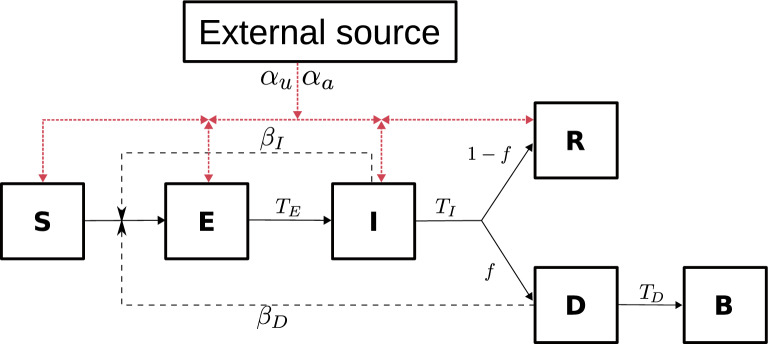
SEIRD and communication model. Compartmental model of infectious disease dynamics and communication. The continuous lines show the transition between epidemiological states, the black dashed lines the infectious dynamics, and the red dashed lines the flow of information between agents and from the external source. Details of the parameters can be found in Table 1.

To introduce spatial homogeneity into our simulation, we randomly distributed agents across the grid, adhering to a uniform distribution. The initial setting of the, factual information and misinformation, quality constant at $q_{(a,u)i}(0)=100$ for all agents—apart from those initially infected, for whom $q_{ai}(0)=0$—serves a specific purpose. This value effectively represents a baseline of minimal factual information or misinformation, chosen because it yields an awareness-information/unawareness-information state $r_{(a,u)i}$ almost negligible when applied to equation $r_{(a,u)i}=\rho ^{q_{(a,u)i}}$, for $\rho _{(a,u)} \in [0.1,0.9]$. This ensures that at the start, the majority of agents possess virtually no awareness or unawareness about the pandemic, with $r_{(a,u)i} \in [10^{-100}, 2\times 10^{-5}]$, setting a uniform stage for the spread and impact of information as the simulation progresses. The compartmental model is shown in Fig 1.

In our simulation’s initial configuration, 100 agents are designated as infected, $Q_i = I$, to catalyze the epidemiological dynamics from the outset, while the rest are susceptible, $Q_i = S$. This initial infection number was chosen to strike a balance between a realistic start to an outbreak and computational manageability, ensuring dynamic interactions between health states and information flow can emerge naturally. The model’s capacity for agents to concurrently hold factual information and misinformation introduces nuanced dynamics that reflect the complexities of real-world information consumption and belief formation. For example, an agent might be aware of the need for handwashing (factual information) while also believing in unproven treatments (misinformation) such as bleach injection, affecting their behavior in ways that are not straightforwardly predictable^54^.

Initial infected agents were randomly selected, emphasizing the unpredictable nature of disease spread and ensuring diverse interactions within the simulation environment. This approach, coupled with the distinct starting points for information quality, lays a foundational framework for exploring how factual information, misinformation, and epidemiological states evolve and influence one another over time.

## Results and discussion

As a starting point, we explore a scenario without misinformation, with the inclusion of a component denoting preventive behavior $\gamma _a$, acting over the infection rate $\beta _I$. This inspection allows us to examine more closely how factual information alone influences the spread of the disease and the agents’ responses. Subsequently, we extend our investigation to a more complex scenario that incorporates misinformation originating from a central entity and circulating via *word-of-mouth*, mirroring real-world conditions where factual information and misinformation coexist and compete. By comparing these two scenarios, we aim to uncover the effects of misinformation on the epidemic’s dynamics and the population’s behavior, highlighting the significance of the preventive and harmful behavior $\gamma _{a}$ and $\gamma _{u}$, respectively, when navigating an environment riddled with factual information and misinformation.

### A system with factual information

**Fig. 2:**
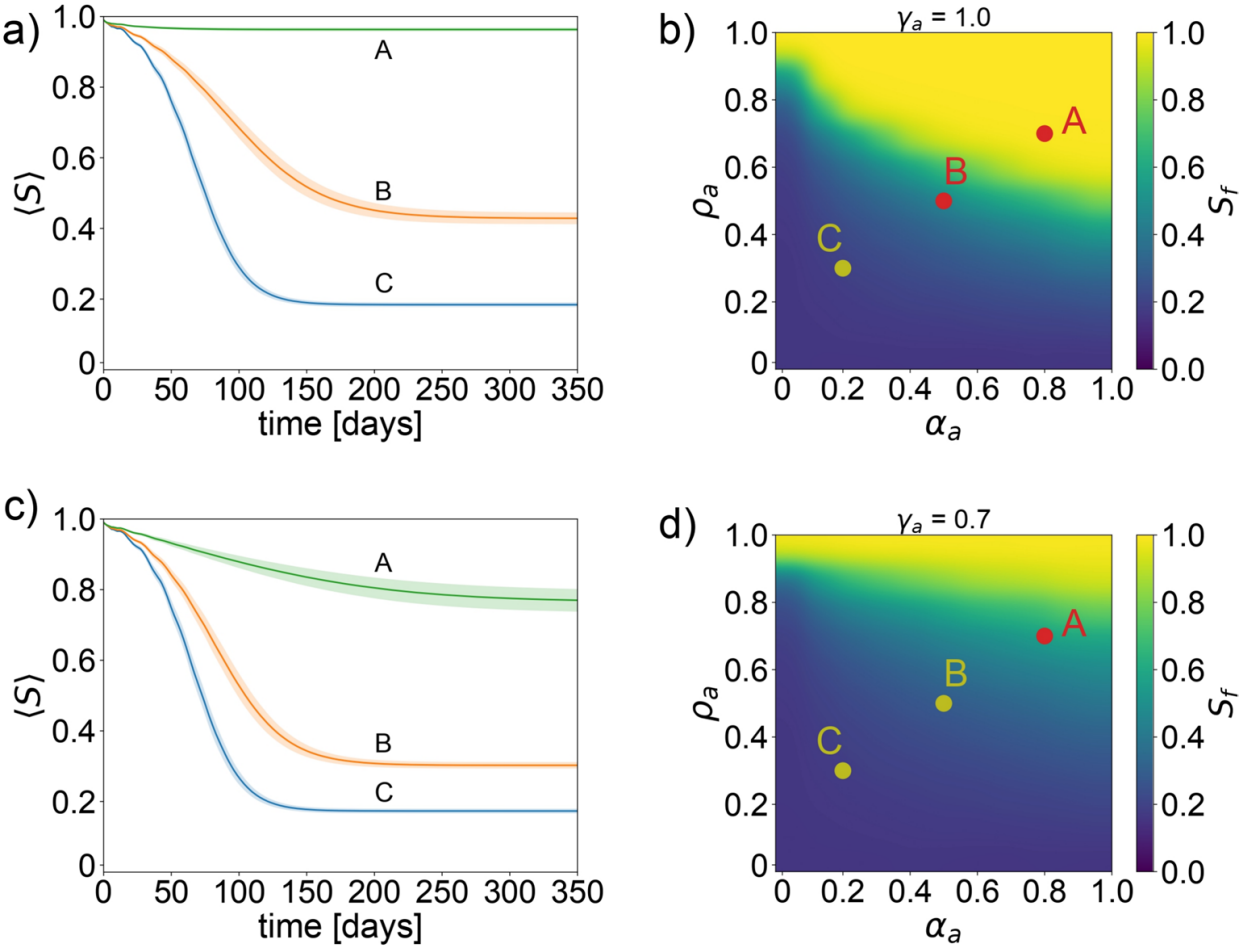
System with factual information. Exploration of the model for a scenario where there is only factual information. Panel (**a**), examines the time evolution of the susceptible agent ratio across varying levels of awareness decay constant $\rho _a$ and the ratio of informed individuals $\alpha _a$, with a constant preventive behavior parameter $\gamma _a=1$. Panel (**b**), offers a density plot that visualizes the final ratio of susceptible agents at the simulation’s conclusion, correlating with different values of $\rho _a$ and $\alpha _a$, under the assumption of preventive behavior $\gamma _a=1$. Panel (**c**), revisits the time evolution of the susceptible agent ratio for an array of $\rho _a$ and $\alpha _a$ values, this time with the preventive behavior parameter adjusted to $\gamma _a=0.7$. Panel (**d**), presents a density plot detailing the final ratio of susceptible agents, examining the impacts of varying $\rho _a$ and $\alpha _a$, with preventive behavior set at $\gamma _a=0.7$.

For our simulations, as detailed in Fig. 2, we employed the parameters delineated in Table 2, concentrating solely on the dissemination and ramifications of factual information. We proceed under the premise that the behaviors of individuals are in perfect concordance with the information they are exposed to, thereby adhering strictly to the recommended preventive actions.

**Table 2:** Parameters for different simulations (different parameters). In Fig. 2, setting $\alpha _u=0$ denotes the absence of misinformation, effectively negating harmful behavior denoted by $\gamma _u$. Conversely, Figs. 3 and 4 introduce misinformation to the simulations. Each parameter set was executed 100 times.

	$\gamma _a$	$\gamma _u$	$\alpha _a$	$\alpha _u$	$\rho _a$	$\rho _u$
Figure 2	0.7, 1.0	0	Variable	0	Variable	0
Figure 3	1.0	1.0	1.0, 0.5	1.0	Variable	Variable
Figure 4	1.0	1.0	1.0	1.0, 0.1	Variable	Variable

The results, depicted in Fig. 2B and further elaborated in Supplementary Figure S1, illustrate how variations in the decay constant of factual information $\rho _a$ and the preventive behavior coefficient $\gamma _a$ affect the simulation outcomes. The color gradient in the figure indicates the final number of susceptible individuals $S_f$, with higher values represented in yellow, signaling more effective epidemic control.

Interestingly, even at the highest level of preventive behavior ($\gamma _a=1.0$), the area within which the disease is effectively halted (indicated in yellow) is relatively small compared to the regions where the disease exerts a greater impact (shown in blue). This discrepancy becomes more pronounced as the preventive behavior level decreases to $\gamma _a=0.7$, still considered significantly proactive.

A critical observation from our analysis is that the yellow region, denoting effective disease control, emerges only when both the ratio of informed individuals with factual information $\alpha _a$ and the awareness decay constant $\rho _a$ are high. This indicates that for a population to derive benefit, it is crucial that a substantial proportion remains well-informed and that the information retains its behavioral influence over time. Conversely, a decrease in either $\alpha _a$ or $\rho _a$ invariably leads to adverse outcomes for the population. These findings emphasize the vital importance of maintaining a high ratio of people informed with factual information $\alpha _a$ and fostering effective preventive behaviors $\gamma _a$. Given the uncertain behavior of $\rho _a$ in real-world scenarios, prioritizing high values for $\alpha _a$ and $\gamma _a$ could significantly enhance our ability to protect populations during epidemics.

This analysis reveals the essential role of reliable information and behavior compliance in controlling epidemic spread, offering valuable insights into optimizing public health responses.

### A system with both factual information and misinformation

**Fig. 3:**
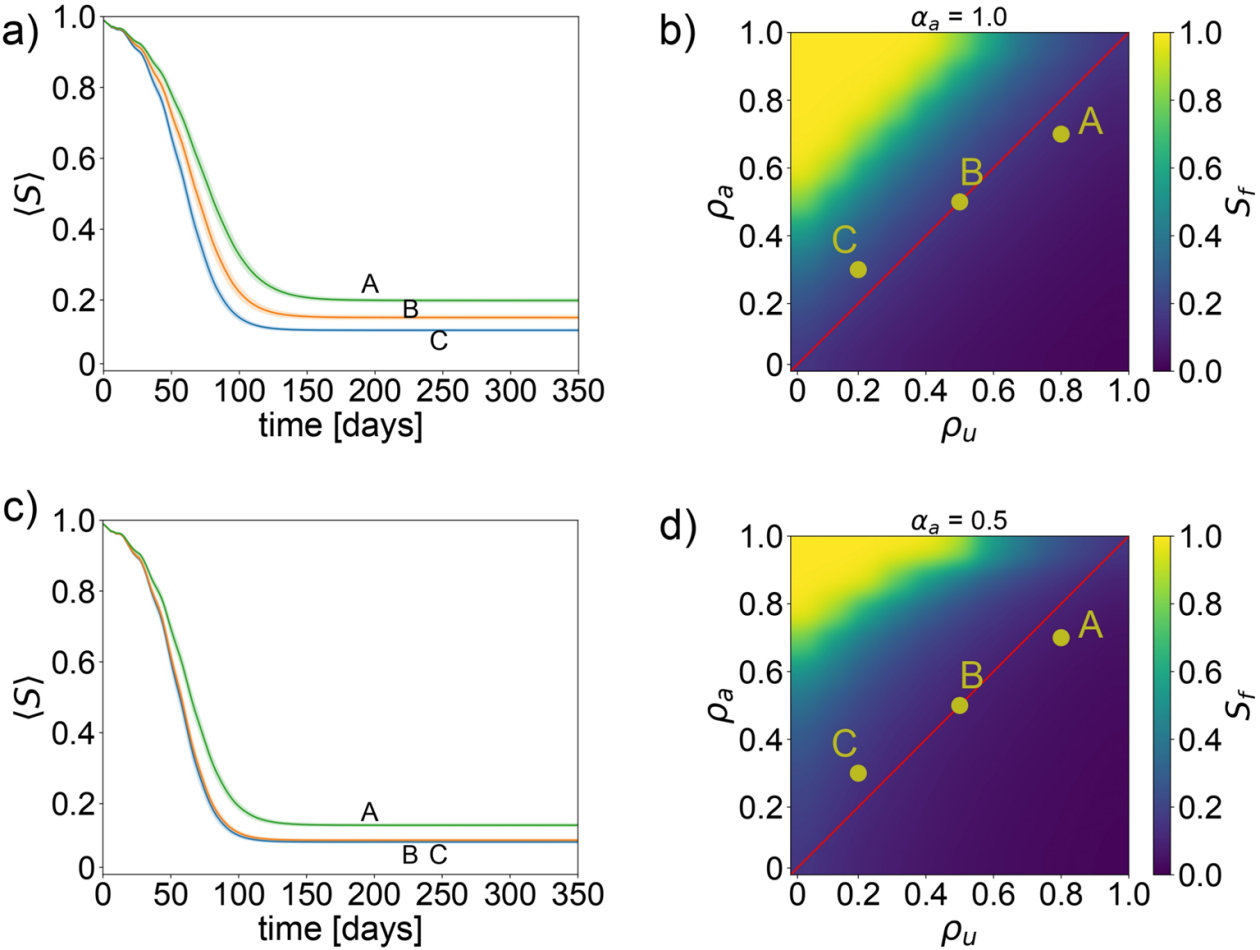
System with variably factual information and constant misinformation We analyze scenarios where the proportion of individuals informed with misinformation is fixed at 1.0, while the proportion of those receiving factual information varies from 1.0 to 0.5. We denote different configurations of $\rho _{u,a}$—the parameters representing misinformation and awareness decay constant, respectively—as points A, B, and C, with $A(\rho _u, \rho _a)=(0.8, 0.7)$, $B(\rho _u, \rho _a)=(0.5, 0.5)$, and $C(\rho _u, \rho _a)=(0.2, 0.3)$. The harmful behavior adjustment factor is consistently set at $\gamma _{a,u}=1$. Panel (**a**), illustrates the time evolution of the susceptible agent ratio for configurations A, B, and C. Panel (**b**), displays a density plot showing the final susceptible agent ratio at the simulation’s conclusion across varying levels of $\rho _a$ and $\rho _u$. These panels account for a scenario where the population is informed with a mix of factual information and misinformation in equal proportions (1.0 each). Panel (**c**), repeats the time evolution analysis of the susceptible agent ratio for points A, B, and C, under modified conditions. Panel (**d**), presents a corresponding density plot for the final susceptible agent ratio, considering different values of $\rho _a$ and $\rho _u$. Here, the ratio of the population informed with factual information is reduced to 0.5, maintaining the misinformation level at 1.0.

For simplicity, in this section, we initially assume that the dissemination rates of both factual information and misinformation among individuals are equivalent. The results of this augmented scenario are presented in Fig. 3, where, in contrast to Fig. 2, the y-axis now represents the awareness decay constant $\rho _a$, and the x-axis denotes the misinformation decay constant $\rho _u$.

Examining the temporal evolution of our SEIRD model through Fig. 3a, we observe three distinct simulations—labelled A, B, and C—each characterized by unique pairs of $\rho _a$ and $\rho _u$ values: $A(\rho _a, \rho _u)=(0.7, 0.8)$, $B(\rho _a, \rho _u)=(0.5, 0.5)$, and $C(\rho _a, \rho _u)=(0.3, 0.2)$. Interestingly, these simulations reveal a comparable final count of susceptible individuals, suggesting minimal variance in epidemic size across these parameter sets when $100\%$ of the population receives factual information.

This observation is further visualized in Fig. 3b, which displays a density plot of the final number of susceptible individuals. Notably, the region (depicted in yellow) indicating a high final number of susceptibles—corresponding to a low epidemic impact—is relatively small, even when the factual information dissemination rate is at 1.0. This indicates that universal dissemination of factual information alone does not significantly mitigate the spread of the epidemic.

To more closely align our simulation with real-world dynamics, we include a red line to represent scenarios where $\rho _a = \rho _u$. Given literature suggesting that misinformation tends to persist longer within populations^1^, we infer that realistic scenarios likely feature $\rho _a < \rho _u$. Consequently, simulations falling beneath this red line are considered more plausible, implicitly excluding the optimistic yellow region from real-world applicability. This reinforces the conclusion that, despite the global dissemination of factual information, the potential to control the epidemic remains limited when misinformation is present. Figure 3c,d further illustrate the system’s behavior with only $50\%$ of the population receiving factual information. A comparison with the $100\%$ informed, with factual information, scenario emphasizes that a higher factual information dissemination rate marginally improves outcomes. Yet, similar to the fully informed case, this scenario predominantly resides above the red line, suggesting its improbability in a real-world context. This analysis accentuates the complex challenges in achieving effective epidemic control through factual information dissemination alone, especially in the presence of competing misinformation. For a comprehensive view of how varying degrees of factual information dissemination affect the simulation—from a ratio of 0.0 (no factual information) to 1.0 (everyone is informed with factual information)—please refer to Supplementary Figure S2.

**Fig. 4:**
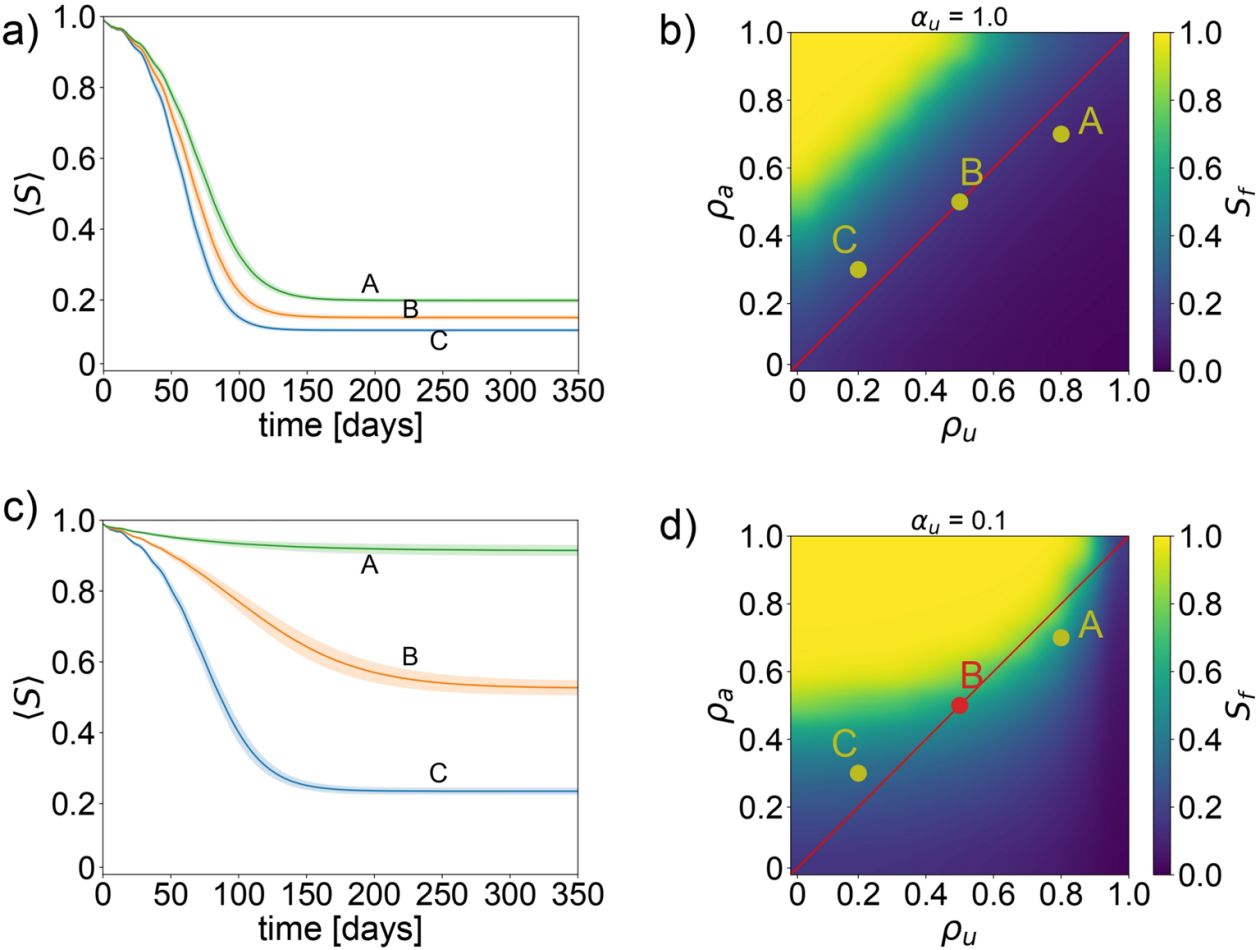
System with constant factual information and variable misinformation. In this exploration of the model, we vary the ratio of individuals informed with misinformation from 1.0 to 0.1. Scenarios are illustrated through points A, B, and C, each representing distinct values of $\rho _{u,a}$: $A(\rho _u, \rho _a)=(0.8, 0.7)$, $B(\rho _u, \rho _a)=(0.5, 0.5)$, and $C(\rho _u, \rho _a)=(0.2, 0.3)$. Throughout, we maintain the behavior adjustment parameter at $\gamma _{a,u}=1$. Panel (**a**) details the time evolution of the susceptible agent ratio at the specified points A, B, and C. Panel (**b**) displays a density plot showing the final ratio of susceptible agents at the end of the simulation, examining various levels of awareness decay constant $\rho _a$ and misinformation decay constant $\rho _u$. In this scenario, the population is equally informed with both factual information and misinformation (ratio of 1.0 each). Panel (**c**) again observes the time evolution of the susceptible agent ratio for points A, B, and C, under modified information distribution. Panel (**d**) provides a density plot of the final susceptible agent ratio, exploring different values $\rho _a$ and $\rho _u$. This analysis adjusts the ratio of the population informed with factual information to 1.0, while reducing the misinformation spread to a ratio of 0.1.

Building on our earlier analysis, we now contrast two systems with a focus on adjusting the proportion of individuals receiving misinformation, while maintaining constant exposure to factual information across the entire population (fixed ratio at 1.0, indicating that everyone is exposed to factual information). This comparative study is visualized in Fig. 4, which notably illustrates the high impact of the misinformation, observable by comparing panel b and d of Fig. 4. In Fig. 4b (is the same of Fig. 3b) we have all the population misinformed, resulting in a yellow area above the red line, that indicates the epidemic has almost no chance to be stopped. In the second scenario shown in Fig. 4c, d, just $10\%$ of the people are misinformed, but nevertheless, the yellow area still remains above the red line, despite increasing its area. We must recall that the parameters below the red line, as previously discussed, represent a likely realistic condition where the misinformation decay constant $\rho _u$ is bigger than the awareness decay constant $\rho _a$ (people remember more misinformation). This stipulation further diminishes the likelihood of optimistic scenarios, reinforcing the challenge of achieving meaningful public health outcomes even under a low amount of misinformation influence. For a comprehensive view of how varying degrees of misinformation dissemination affect the simulation—from a ratio of 0.0 (no misinformation) to 1.0 (everyone is misinformed)—please refer to Supplementary Figure S3. This extended analysis provides deeper insights into the dynamics at play and highlights the critical challenge posed by misinformation in managing public health responses effectively.

### Exploration of the solution landscape through dimensional reduction

In the previous section, we conducted simulations varying certain parameters ($\alpha _a$, $\alpha _u$, $\rho _a$, $\rho _u$) while keeping others constant ($\gamma _a$, $\gamma _u$), gaining preliminary insights into our model’s behavior and its potential real-world implications. Building upon this, we embark on a comprehensive exploration of the parameter space through a grid search to uncover the full spectrum of possible model behaviors. To manage the complexity of our multidimensional data—originating from a simulation space defined by six variable parameters—we employ the UMAP (Uniform Manifold Approximation and Projection) algorithm^55^ for dimensionality reduction and clustering. This process transforms our 7-dimensional vector: ($\gamma _a$, $\gamma _u$, $\alpha _a$, $\alpha _u$, $\rho _a$, $\rho _u$, $S_f$), where $S_f$ denotes the final count of susceptible individuals, in a more interpretable form, now representing each simulation vector as a two-dimensional vector, which is mapped in the UMAP plot.

**Fig. 5:**
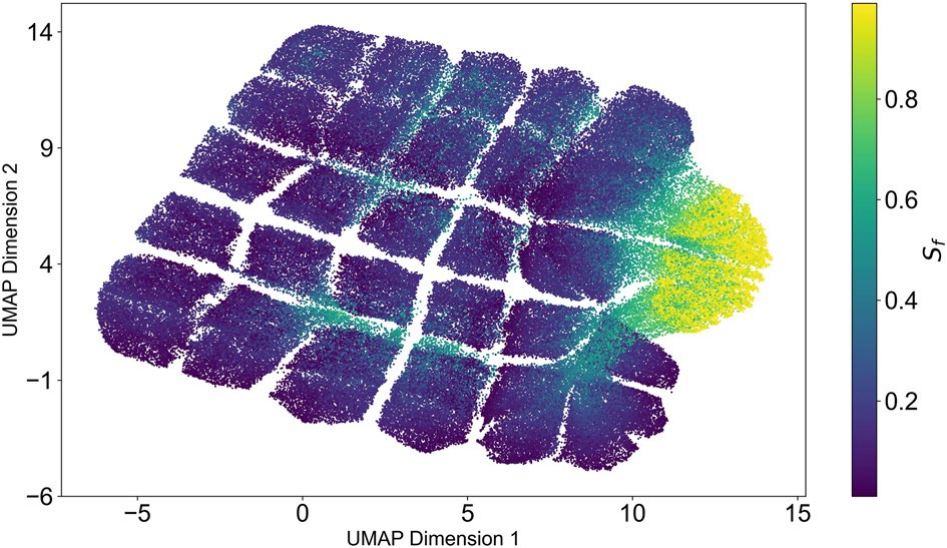
UMAP visualization. Exploration of the model across all parameter combinations. Each point on the visualization marks the final state of susceptible individuals from a single simulation. The yellow region shows an area where the final counts of susceptible individuals in the simulation are notably high.

The UMAP visualization, depicted in Fig. 5, reveals a critical observation: the yellow region, indicative of scenarios where the epidemic impact is minimal or halted, occupies a notably small portion of the solution landscape. This suggests that the majority of parameter combinations result in unfavorable outcomes, characterized by a low final number of susceptible individuals $S_f$. A closer examination (see Sup. Figure S4) of this constrained yellow area reveals that successful outcomes correlate with higher awareness decay constants (implying that factual information persists longer in the population) and lower misinformation decay constants (indicating a shorter lifespan for misinformation). Further analysis highlights that scenarios conducive to positive public health outcomes are associated with a stronger emphasis on preventive behavior (higher $\gamma _a$) over harmful behavior (lower $\gamma _u$). Despite this, the prevailing real-world trend, that misinformation tends to circulate more extensively than accurate information (manifested as $\rho _u > \rho _a$), renders these optimal scenarios challenging to achieve.

**Fig. 6:**
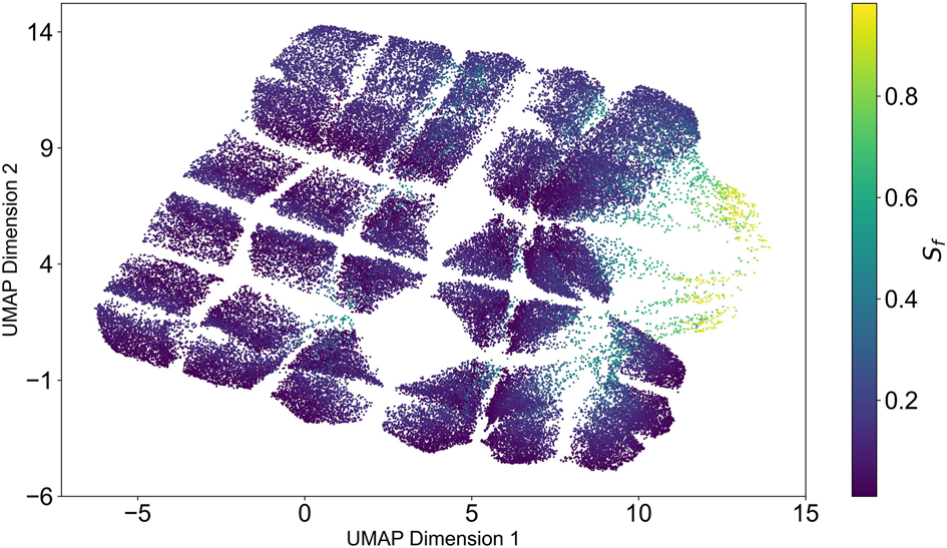
UMAP visualization when $\rho _u>\rho _a$ (bigger impact of misinformation, according to literature) Exploration of the model across all parameter combinations, focusing specifically on scenarios where $\rho _a < \rho _u$. Each point on the visualization represents the final state of susceptible individuals from a single simulation. The yellow region shows an area where the final counts of susceptible individuals in the simulation are notably high.

In an additional visualization presented in Fig. 6, we focus solely on simulations where $\rho _u > \rho _a$, as suggested by literature^1,^ that misinformation often outlasts factual information. This constraint significantly reduces the number of scenarios leading to the epidemic’s cessation, underscoring the formidable obstacle posed by the proliferation of misinformation. We also look closer at this analysis (see Sup. Figure S5), and we can observe that the remaining numbers of scenarios in which the final number of susceptible is high are related to a low value of harmful behavior (low $\gamma _u$).

## Conclusion

This study has embarked on a detailed examination of the interplay between factual information and misinformation within the context of infectious disease outbreaks, with a particular lens on EVD. By leveraging a computational modeling approach that combines an ABM with a SEIRD compartmental model, we have studied the complex dynamics that govern the spread of infectious diseases alongside the propagation of both factual information and misinformation.

To do so, we introduced a comprehensive model integrating the dissemination of both infectious diseases and information among a population. This approach allowed us to investigate the pivotal role of information dissemination in combating the spread of infectious diseases, focusing on the impact of factually informed versus misinformed segments within the population. Our findings underscore the potential of strategic information delivery to effectively counter disease proliferation, drawing a parallel to the concept of vaccination in controlling outbreaks. Importantly, we propose that the efficacy of information in curbing disease spread is significantly influenced by the duration for which the information remains relevant within the community, highlighting the critical role of information decay over time.

To the best of our knowledge, the relationship between epidemics and the proliferation of misinformation remains largely uncharted in scholarly research, although a few studies^56–61^ begin to address this complex interplay. In fact, Brainard et al. utilized an ABM to simulate the dissemination of misinformation during a norovirus outbreak, which highlighted the significant threat posed by misinformation in the context of an epidemic^59^. A distinct aspect of our study is the incorporation of a temporal decay in the information assimilated by individuals, a feature not considered in the aforementioned research. Moreover, our analysis revealed the harmful effects of misinformation on public health outcomes, emphasizing the need for robust strategies to mitigate its harmful influence during the spread of an infectious disease.

Our research illuminates the profound influence misinformation can exert on escalating the transmission and severity of infectious diseases. The introduction of even minor amounts of misinformation can significantly amplify disease spread, primarily due to its capacity to encourage harmful behaviors, such as disregarding health advisories and adopting infection-prone practices. In contrast, the propagation of factual information serves as a bulwark in curtailing the impact of outbreaks by fostering preventive behaviors. Nevertheless, the potency of factual information is intricately linked to its dissemination scope and the durability of its behavioral influence. As we move forward, facing new pandemic threats^62–64^, it is imperative to build upon this foundational work, expanding our understanding and developing innovative approaches to safeguard public health in the face of emerging threats.

Of note, the analytical outcomes of our model highlight that optimal public health scenarios, marked by a large reservoir of susceptible individuals at the epidemic’s conclusion, are attainable with higher rates of awareness decay and lower rates of misinformation decay. Such findings underline the essential nature of enduring factual information and vastly combating misinformation in epidemic control. Yet, the challenge posed by the pervasive spread and lasting presence of misinformation, as opposed to factual information, presents a formidable obstacle. In our favor, it is worth noting that, in the real world, misinformation typically represents a smaller share of available information, as noted by Allen et al.^65^, a scenario that our model also explores.

Given the challenges associated with mitigating the spread of misinformation, coupled with the insights gleaned from our model and the relevant literature^66,67^, we suggest adopting techniques often utilized in the dissemination of misinformation, such as amplifying emotional engagement, to improve the spread and retention of factual information. Specifically, this approach aims to increase the awareness decay constant $\rho _a$ (raise the red line on our density plots, Figs. 3 and 4), thereby enhancing the effectiveness of accurate information dissemination.

By adopting methods that promote a longer decay period for awareness, we can aspire to shift the balance towards more favorable outcomes, as suggested by our model’s predictions. This approach shows a suitable direction for public health communication strategies, aiming to harness the power of emotional resonance to effectively counteract the spread of misinformation.

As a whole, our work signals the dire consequences of misinformation, urging the adoption of sophisticated communication strategies to enhance factual information spread and fortify societal defenses against both misinformation and infectious diseases. As the global community braces for future pandemics, it becomes imperative to expand upon these findings, crafting innovative strategies to protect public health against the twin threats of disease and misinformation.

## Supplementary Information

Supplementary Information.

## Data Availability

All data generated and analysed in this study, the simulation model implemented in NetLogo 6.1.1, and all scripts used for data processing and statistical analysis are publicly available in the GitHub repository https://github.com/alejob/paper_disease_misinformation

## References

[1] Vosoughi, S., Roy, D. & Aral, S. The spread of true and false news online. *Science* **359**(6380), 1146–1151 (2018). 29590045 10.1126/science.aap9559

[2] Lazer, D. M. et al. The science of fake news. *Science* **359**(6380), 1094–1096 (2018). 29590025 10.1126/science.aao2998

[3] Ferguson, N. Capturing human behaviour. *Nature* **446**(7137), 733–733 (2007). 17429381 10.1038/446733a

[4] WHO, World Health Organization, infodemic, https://www.who.int/health-topics/infodemic#tab=tab_1, accessed: 2021-10-05 (2021).

[5] Gallotti, R., Valle, F., Castaldo, N., Sacco, P. & Domenico, M. Assessing the risks of ‘infodemics’ in response to COVID-19 epidemics. *Nat. Hum. Behav.* **4**(12), 1285–1293 (2020). 33122812 10.1038/s41562-020-00994-6

[6] Tiwari, S., Kanchan, S., Subash, N. R. & Bajpai, P. Prevalence of authentic versus false information in general population during covid 19 pandemic. *Indian J. Prevent. Social Med.* **51**(2), 61–71 (2020).

[7] Linden, S., Roozenbeek, J. & Compton, J. Inoculating against fake news about covid-19. *Front. Psychol.* **11**, 2928 (2020). 10.3389/fpsyg.2020.566790 PMC7644779 33192844

[8] Y. M. Rocha, G. A. de Moura, G. A. Desidério, C. H. de Oliveira, F. D. Lourenço, L. D. de Figueiredo Nicolete, The impact of fake news on social media and its influence on health during the covid-19 pandemic: A systematic review, Journal of Public Health 1–10 (2021). 10.1007/s10389-021-01658-z PMC8502082 34660175

[9] Campbell, E. M. & Salathé, M. Complex social contagion makes networks more vulnerable to disease outbreaks. *Sci. Rep.*10.1038/srep01905 (2012). 10.1038/srep01905 PMC3664906 23712758

[10] Khan, A., Naveed, M., Ahmad, M. D. E. & Imran, M. Estimating the basic reproductive ratio for the ebola outbreak in liberia and sierra leone. *Infect. Diseases Poverty.*10.1186/s40249-015-0043-3 (2015). 10.1186/s40249-015-0043-3 PMC4347917 25737782

[11] Gonsalves, G. & Staley, P. Panic, paranoia, and public health—The aids epidemic’s lessons for ebola. *N. Engl. J. Med.* **371**(25), 2348–2349 (2014). 25372947 10.1056/NEJMp1413425

[12] Umeora, O. U. et al. Ebola viral disease in Nigeria: The panic and cultural threat. *Afr. J. Med. Health Sci.* **13**(1), 1 (2014).

[13] J. Hageman, C. Hazim, K. Wilson, P. Malpiedi, N. Gupta, S. Bennett, A. R. Kolwaite, A. J. Tumpey, K. J. Brinsley-Rainisch, B. E. Christensen, C. Gould, A. Fisher, M. Jhung, D. Hamilton, K. Moran, L. Delaney, C. Dowell, M. Bell, A. Srinivasan, M. Schaefer, R. Fagan, N. Adrien, N. Chea, B. J. Park, Infection prevention and control for ebola in health care settings - west africa and united states., MMWR supplements 65 3, 50–6. 10.15585/mmwr.su6503a8 (2016). 10.15585/mmwr.su6503a8 27390018

[14] Vinck, P., Pham, P., Bindu, K. K., Bedford, J. & Nilles, E. Institutional trust and misinformation in the response to the 2018–19 Ebola outbreak in North Kivu, Dr Congo: A population-based survey., The Lancet. *Infectious diseases* **19 5**, 529–536. 10.1016/S1473-3099(19)30063-5 (2019). 10.1016/S1473-3099(19)30063-5 30928435

[15] Sell, T., Hosangadi, D. & Trotochaud, M. Misinformation and the us ebola communication crisis: Analyzing the veracity and content of social media messages related to a fear-inducing infectious disease outbreak. *BMC Public Health.*10.1186/s12889-020-08697-3 (2020). 32375715 10.1186/s12889-020-08697-3 PMC7202904

[16] Marzi, A. & Feldmann, H. Ebola virus vaccines: An overview of current approaches. *Expert Rev. Vaccines* **13**, 521–531. 10.1586/14760584.2014.885841 (2014). 24575870 10.1586/14760584.2014.885841 PMC4785864

[17] Dhama, K. et al. Advances in designing and developing vaccines, drugs, and therapies to counter ebola virus. *Front. Immunol.*10.3389/fimmu.2018.01803 (2018). 30147687 10.3389/fimmu.2018.01803 PMC6095993

[18] Kermack, W. O. & Mckendrick, À. G. A contribution to the mathematical theory of epidemics. *Proc. R. Soc. A Math. Phys. Eng. Sci*. **115**, 700–721 (1927).

[19] Huang, G. & Takeuchi, Y. Global analysis on delay epidemiological dynamic models with nonlinear incidence. *J. Math. Biol.* **63**, 125–139. 10.1007/s00285-010-0368-2 (2011). 20872265 10.1007/s00285-010-0368-2

[20] Li, M. Y. & Muldowney, J. Global stability for the seir model in epidemiology. *Math. Biosci.* **125 2**, 155–64. 10.1016/0025-5564(95)92756-5 (1995). 7881192 10.1016/0025-5564(95)92756-5

[21] T. Veloz, P. Maldonado, S. Ropert, C. Ravello, S. Mora, A. Barrios, T. Villaseca, C. Valdenegro, T. Perez-Acle, On the interplay between mobility and hospitalization capacity during the covid-19 pandemic: The seirhud model (2020). arXiv:2006.05357.

[22] Britton, T., Ball, F. & Trapman, P. A mathematical model reveals the influence of population heterogeneity on herd immunity to sars-cov-2. *Science (New York, N.y.)* **369**, 846–849. 10.1126/science.abc6810 (2020). 32576668 10.1126/science.abc6810 PMC7331793

[23] M. G. M. Gomes, R. M. Corder, J. G. King, K. e. Langwig, C. Souto-Maior, J. Carneiro, G. Gonçalves, C. Penha-Gonçalves, M. U. Ferreira, R. Águas, Individual variation in susceptibility or exposure to sars-cov-2 lowers the herd immunity threshold, Journal of Theoretical Biology 540 (2020) 111063 – 111063. 10.1016/j.jtbi.2022.111063. 10.1016/j.jtbi.2022.111063 PMC8855661 35189135

[24] Kiss, I. Z., Cassell, J., Recker, M. & Simon, P. L. The impact of information transmission on epidemic outbreaks. *Math. Biosci.* **225**(1), 1–10 (2010). 19948177 10.1016/j.mbs.2009.11.009

[25] Granell, C., Gómez, S. & Arenas, A. Dynamical interplay between awareness and epidemic spreading in multiplex networks. *Phys. Rev. Lett.* **111**(12), 128701 (2013). 24093306 10.1103/PhysRevLett.111.128701

[26] Granell, C., Gómez, S. & Arenas, A. Competing spreading processes on multiplex networks: Awareness and epidemics. *Phys. Rev. E* **90**(1), 012808 (2014). 10.1103/PhysRevE.90.012808 25122343

[27] Funk, S., Gilad, E. & Jansen, V. Endemic disease, awareness, and local behavioural response. *J. Theoret. Biol.* **264**(2), 501–509 (2010). 20184901 10.1016/j.jtbi.2010.02.032

[28] Bernardin, A., Martínez, A. J. & Perez-Acle, T. On the effectiveness of communication strategies as non-pharmaceutical interventions to tackle epidemics. *PloS One* **16**(10), e0257995 (2021). 34714848 10.1371/journal.pone.0257995 PMC8555801

[29] Funk, S., Gilad, E., Watkins, C. & Jansen, V. A. The spread of awareness and its impact on epidemic outbreaks. *Proc. Natl. Acad. Sci.* **106**(16), 6872–6877 (2009). 19332788 10.1073/pnas.0810762106 PMC2672559

[30] Chen, F. Modeling the effect of information quality on risk behavior change and the transmission of infectious diseases. *Math. Biosci.* **217 2**, 125–33. 10.1016/j.mbs.2008.11.005 (2009). 19059272 10.1016/j.mbs.2008.11.005

[31] Samanta, S. & Chattopadhyay, J. Effect of awareness program in disease outbreak—A slow-fast dynamics. *Appl. Math. Comput.* **237**, 98–109. 10.1016/j.amc.2014.03.109 (2014).

[32] Funk, S., Salathé, M. & Jansen, V. A. Modelling the influence of human behaviour on the spread of infectious diseases: A review. *J. R. Soc. Interface* **7**(50), 1247–1256 (2010). 20504800 10.1098/rsif.2010.0142 PMC2894894

[33] Zhang, H., Xie, J.-R., Tang, M. & Lai, Y. Suppression of epidemic spreading in complex networks by local information based behavioral responses. *Chaos.*10.1063/1.4896333 (2014). 25554026 10.1063/1.4896333 PMC7112481

[34] Kremer, J. R. & Muller, C. P. Measles in Europe—There is room for improvement. *Lancet* **373**(9661), 356–358 (2009). 19131098 10.1016/S0140-6736(08)61850-4

[35] Abruzzi, W. Measles—A serious pediatric disease. *J. Pediatr.* **64**(5), 750–752 (1964). 14149010 10.1016/s0022-3476(64)80624-7

[36] Berger, J. & Milkman, K. L. What makes online content viral?. *J. Marketing Res.* **49**(2), 192–205 (2012).

[37] ABC, Nigerian ebola hoax results in two deaths, *ABC News*, retrieved from https://abcnews.go.com/Health/nigerian-ebola-hoax-results-deaths/story?id=25842191 (Sep. 2014).

[38] WHO Ebola Response Team. Ebola virus disease in west Africa-The first 9 months of the epidemic and forward projections. *N. Engl. J. Med.* **2014**(371), 1481–1495 (2014). 10.1056/NEJMoa1411100 PMC4235004 25244186

[39] Victory, K. R., Coronado, F., Ifono, S. O., Soropogui, T. & Dahl, B. A. Ebola transmission linked to a single traditional funeral ceremony—Kissidougou, Guinea, December, 2014–January 2015. *MMWR. Morbidity Mortality Weekly Rep.* **64**(14), 386 (2015). PMC5779538 25879897

[40] Weitz, J. S. & Dushoff, J. Modeling post-death transmission of ebola: Challenges for inference and opportunities for control. *Sci. Rep.* **5**, 8751 (2015). 25736239 10.1038/srep08751 PMC4348651

[41] Althaus, C. L. Estimating the reproduction number of ebola virus (ebov) during the, outbreak in west africa. *PLoS Curr. Outbreaks* **6**(2014), 1–11 (2014). 10.1371/currents.outbreaks.91afb5e0f279e7f29e7056095255b288 PMC4169395 25642364

[42] M. F. Gomes, A. P. y Piontti, L. Rossi, D. Chao, I. Longini, M. E. Halloran, A. Vespignani, Assessing the international spreading risk associated with the 2014 west African Ebola outbreak, PLOS Currents Outbreaks 6 (2014). 10.1371/currents.outbreaks.cd818f63d40e24aef769dda7df9e0da5 PMC4169359 25642360

[43] D. Fisman, E. Khoo, A. Tuite, Early epidemic dynamics of the west african 2014 ebola outbreak: estimates derived with a simple two-parameter model, PLOS Currents Outbreaks 6 (2014). 10.1371/currents.outbreaks.89c0d3783f36958d96ebbae97348d571 PMC4169344 25642358

[44] Dénes, A. & Gumel, A. B. Modeling the impact of quarantine during an outbreak of ebola virus disease. *Infect. Disease Model.* **4**, 12–27 (2019). 30828672 10.1016/j.idm.2019.01.003 PMC6382747

[45] Abo, S. M. et al. Modelling the daily risk of ebola in the presence and absence of a potential vaccine. *Infect. Disease Model.* **5**, 905–917 (2020). 33078134 10.1016/j.idm.2020.10.003 PMC7557810

[46] U. Wilensky, Netlogo. evanston, il: center for connected learning and computer-based modeling, northwestern university (1999).

[47] Sok, J. & Fischer, E. A. Farmers’ heterogeneous motives, voluntary vaccination and disease spread: An agent-based model. *Eur. Rev. Agric. Econ.* **47**(3), 1201–1222 (2020).

[48] E. Hunter, B. Mac Namee, J. Kelleher, An open-data-driven agent-based model to simulate infectious disease outbreaks. *PloS One*. **13**(12), e0208775 (2018). 10.1371/journal.pone.0208775 PMC6300276 30566424

[49] Munkhbat, B. A computational simulation model for predicting infectious disease spread using the evolving contact network algorithm. *Master Theses*. **790** (2019).

[50] Ye, M. & Lyu, Z. Trust, risk perception, and COVID-19 infections: Evidence from multilevel analyses of combined original dataset in china. *Social Sci. Med.* **265**(2020), 113517–113517. 10.1016/j.socscimed.2020.113517 (1982). 10.1016/j.socscimed.2020.113517 PMC7654228 33218890

[51] E. Katz, P. F. Lazarsfeld, E. Roper, Personal influence: The part played by people in the flow of mass communications, Routledge, 2017.

[52] E. M. Rogers, A. Singhal, M. M. Quinlan, Diffusion of innovations, in: An integrated approach to communication theory and research, Routledge, pp. 432–448 (2014).

[53] Daley, D. J. & Kendall, D. G. Stochastic rumours. *IMA J. Appl. Math.* **1**(1), 42–55 (1965).

[54] Rivera, J. M. et al. Evaluating interest in off-label use of disinfectants for COVID-19. *Lancet Digital Health* **2**, e564–e566. 10.1016/S2589-7500(20)30215-6 (2020). 33015597 10.1016/S2589-7500(20)30215-6 PMC7521872

[55] L. McInnes, J. Healy, J. Melville, Umap: Uniform manifold approximation and projection for dimension reduction, arXiv preprint arXiv:1802.03426 (2018).

[56] Sontag, A., Rogers, T. & Yates, C. A. Misinformation can prevent the suppression of epidemics. *J. R. Soc. Interface* **19**(188), 20210668 (2022). 35350880 10.1098/rsif.2021.0668 PMC8965399

[57] Mumtaz, N., Green, C. & Duggan, J. Exploring the effect of misinformation on infectious disease transmission. *Systems* **10**(2), 50 (2022).

[58] Prandi, L. & Primiero, G. Effects of misinformation diffusion during a pandemic. *Appl. Netw. Sci.* **5**, 1–20 (2020). 10.1007/s41109-020-00327-6 PMC7599980 33163618

[59] Brainard, J., Hunter, P. & Hall, I. R. An agent-based model about the effects of fake news on a norovirus outbreak. *Revue D’Épidémiologie Et De Santé Publique* **68**(2), 99–107 (2020). 32037129 10.1016/j.respe.2019.12.001

[60] M. R. DeVerna, F. Pierri, Y.-Y. Ahn, S. Fortunato, A. Flammini, F. Menczer, Modeling the amplification of epidemic spread by misinformed populations, arXiv preprint arXiv:2402.11351 (2024). 10.1038/s44260-025-00038-y PMC11964913 40190382

[61] I. M. Bulai, M. Sensi, S. Sottile, A geometric analysis of the sirs compartmental model with fast information and misinformation spreading, arXiv preprint arXiv:2311.06351 (2023).

[62] Barclay, E. Predicting the next pandemic. *Lancet (London, England)* **372**, 1025–1026. 10.1016/S0140-6736(08)61425-7 (2008). 18810803 10.1016/S0140-6736(08)61425-7 PMC7134898

[63] R. Izurieta, A. Campos, J. Parikh, T. Gardellini, Which plagues are coming next?, Contemporary Developments and Perspectives in International Health Security - Volume 2 [Working Title] (2021). 10.5772/intechopen.96820.

[64] G. Neumann, Y. Kawaoka, Which virus will cause the next pandemic?, Viruses 15 (2023). 10.3390/v15010199. 10.3390/v15010199 PMC9864092 36680238

[65] Allen, J., Howland, B., Mobius, M., Rothschild, D. & Watts, D. J. Evaluating the fake news problem at the scale of the information ecosystem. *Sci. Adv.* **6**(14), eaay3539 (2020). 32284969 10.1126/sciadv.aay3539 PMC7124954

[66] Roozenbeek, J. & Linden, S. Fake news game confers psychological resistance against online misinformation, Palgrave. *Communications* **5**, 1–10. 10.1057/s41599-019-0279-9 (2019).

[67] Lewandowsky, S. & Linden, S. Countering misinformation and fake news through inoculation and prebunking. *Eur. Rev. Social Psychol.* **32**, 348–384. 10.1080/10463283.2021.1876983 (2021).

