# Supplementary material for: Assessing the impact of misinformation during the spread of infectious diseases
Source: Sci Rep. 2025 Oct 6;15:34740. doi: 10.1038/s41598-025-18457-1 (PMC12501366; doi:10.1038/s41598-025-18457-1)
Supplement: Supplementary file 1 — Supplementary Information. [file 41598_2025_18457_MOESM1_ESM.pdf]

Supplementary Information

Supplementary Figures

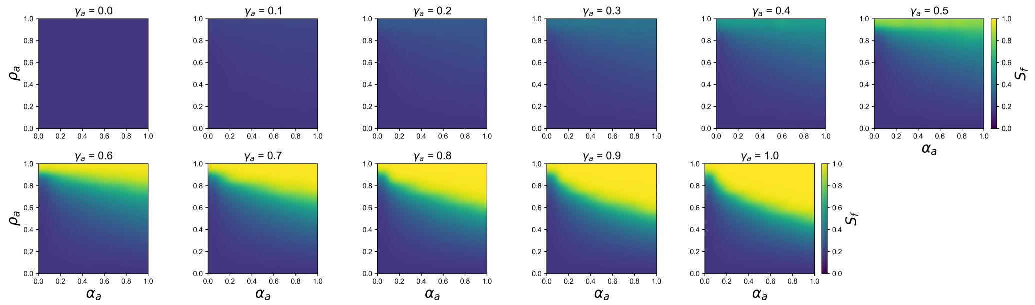

Supplementary Figure S1: **Variation of the preventive behavior in the population.** Density plots depict the final ratio of susceptible individuals in relation to the decay constant ρ_a and the proportion of individuals informed with factual information α_a, across varying levels of preventive behavior γ_a. Figures where $\gamma_a = 0.7$ and $\gamma_a = 1.0$ are the specific plots referenced in Figure 2 of the main text.

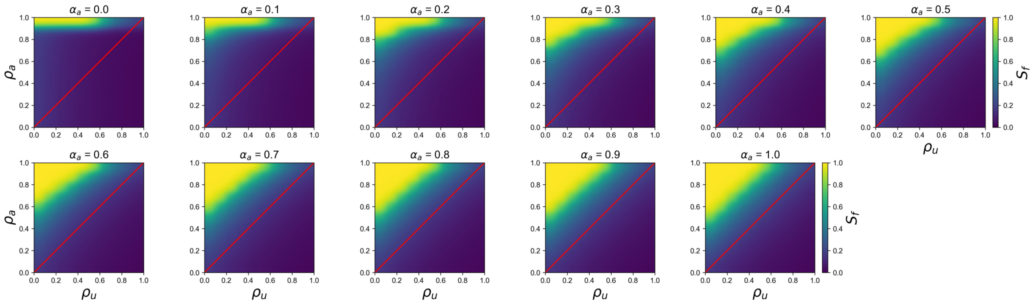

Supplementary Figure S2: **Variation in the proportion of people informed with factual information.** Density plots illustrate the dynamics between the decay constants ρ_a and ρ_u, under conditions where misinformation is pervasive (with 100% of the population exposed to it), and the *ratio of individuals receiving factual information gradually increases*. According to our model and supported by the literature, scenarios falling below the red line ($\rho_a < \rho_u$) depict realistic outcomes. Figures where $\alpha_a = 1.0$ and $\alpha_a = 0.5$ are the specific plots referenced in Figure 3 of the main text.

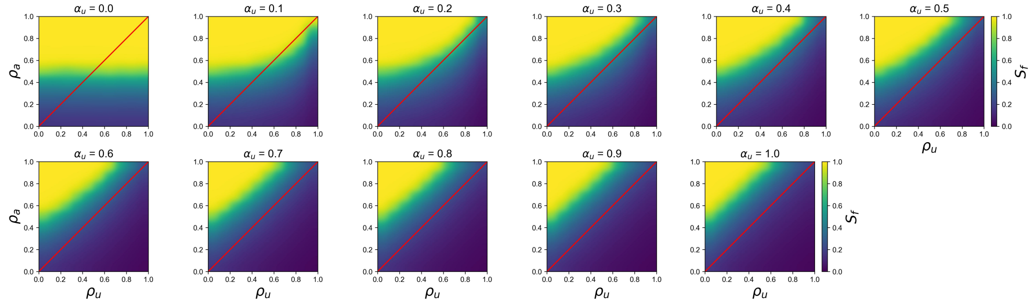

Supplementary Figure S3: **Variation in the proportion of people informed with misinformation.** Density plots reveal the interplay between the decay constants ρ_a and ρ_u, in scenarios where the entire population is initially informed with factual information, but the *ratio of individuals exposed to misinformation gradually increases*. Realistic scenarios, as suggested by our model and corroborated by existing literature, are delineated by values below the red line ($\rho_a < \rho_u$). Figures where $\alpha_u = 1.0$ and $\alpha_u = 0.1$ are the specific plots referenced in Figure 4 of the main text.

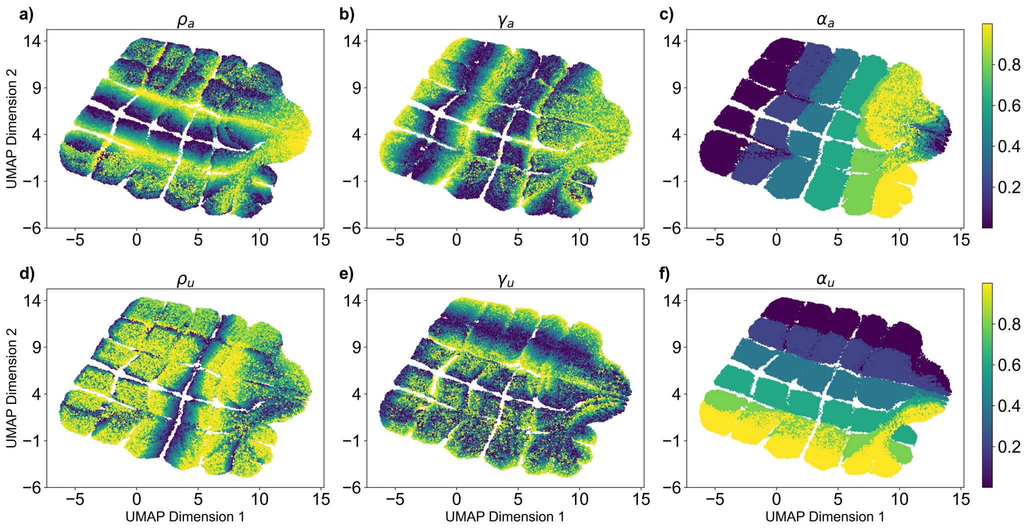

Supplementary Figure S4: **UMAP representation across various parameters.** The UMAP presented in Figure 5 is analyzed further by coloring each point according to different simulation parameters. This results in a series of plots, each representing the same UMAP layout but differentiated by the color-coding of a specific parameter. The parameters and their corresponding plots are as follows: a) awareness decay constant ρ_a, b) preventive behavior parameter γ_a, c) proportion of people informed with factual information α_a, d) misinformation decay constant ρ_u, e) harmful behavior parameter γ_u, and f) proportion of people informed with misinformation α_u. This approach allows for a nuanced visualization of how each parameter influences the overall distribution within the UMAP representation.

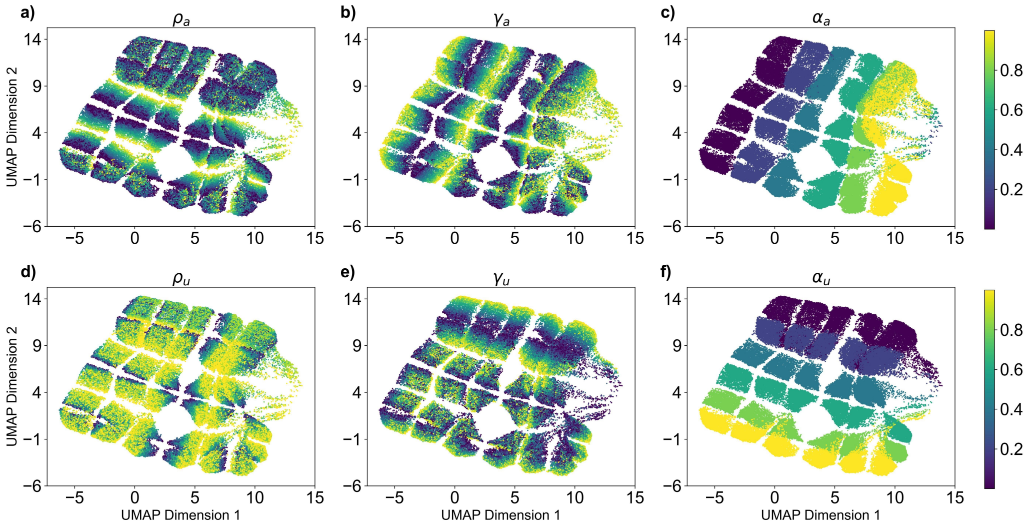

Supplementary Figure S5: **UMAP analyses for $\rho_a < \rho_u$.** In Figure 6, we present a UMAP visualization that is segmented according to various simulation parameters, with each plot showcasing the same UMAP configuration but differentiated by the color representation of each parameter. Specifically, the parameters analyzed include: a) awareness decay constant ρ_a, b) preventive behavior parameter γ_a, c) the proportion of individuals informed with factual information α_a, d) misinformation decay constant ρ_u, e) harmful behavior parameter γ_u, and f) the proportion of individuals informed with misinformation α_u. Importantly, this analysis is restricted to simulations where $\rho_a < \rho_u$, allowing for a focused examination of scenarios that potentially offer realistic insights according to our model and the supporting literature.

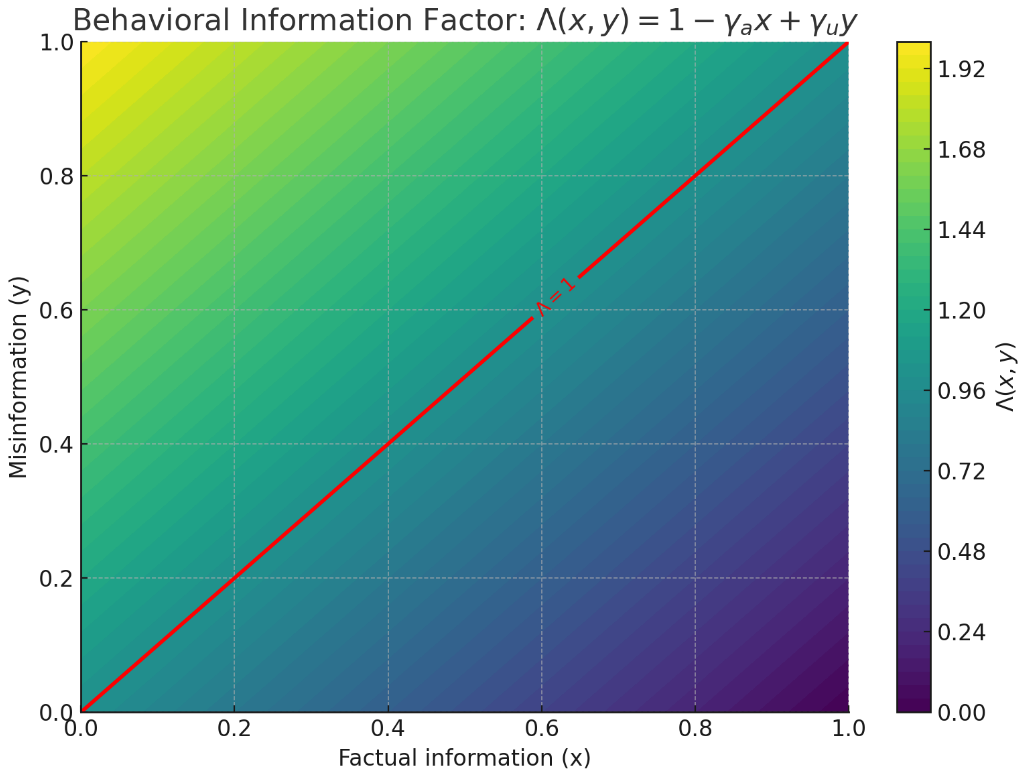

Supplementary Figure S6: **Behavioral-information factor Λ.** Colour shading indicates the local scaling of the transmission rate by Λ: blue regions ($\Lambda < 1$) show net suppression of spread (preventive information dominates), yellow regions ($\Lambda > 1$) show net amplification (misinformation dominates). The solid red line marks the critical contour $\Lambda = 1$, delineating the boundary at which factual and misleading influences exactly balance.

Supplementary tables

Parameter	T_E	T_I	T_D	f	β_I	β_D
Value	11	6	4	0.7	0.25	0.20

Supplementary Table S1: Parameters of the SEIRD model. The values provided correspond to parameters from the 2014-2016 Ebola outbreak in West Africa. Specifically, T_E represents the duration a person remains in the Exposed state before progressing to the Infected state. T_I denotes the time spent in the Infected state before either recovery or death occurs. T_D is the time elapsed from death until burial. The parameter f indicates the fraction of infected individuals who ultimately die. Additionally, β_I and β_D are the infection rates from Infected and Dead individuals to those who are Susceptible, respectively. These parameters were obtained from the study by Weitz et al. (2015).

Parameter	Range	Description
r_{ai}	[0,1]	Awareness-information state
r_{ui}	[0,∞)	Unawareness-information state
ρ_a	[0,1]	Awareness decay constant
ρ_u	[0,1]	Misinformation decay constant
q_{ai}	[0,∞)	Awareness-information quality constant
q_{ui}	[0,∞)	Unawareness-information quality constant
γ_a	[0,1]	Preventive behavior coefficient
γ_u	[0,∞)	Harmful behavior coefficient
α_a	[0,1]	Ratio of informed individuals with factual information
α_u	[0,1]	Ratio of informed individuals with misinformation

Supplementary Table S2: Parameters related to information dynamics in our ABM. we offer a detailed overview of the notations employed in the ABM. It's important to highlight that the harmful behavior coefficient, denoted by γ_u, can potentially reach infinite values. This reflects the reality that harmful behaviors can exacerbate the infection rate exponentially, unlike protective behaviors γ_a, which, by their nature, cannot reduce the infection rate to negative values. This distinction underscores the potentially boundless impact of detrimental actions on the spread of an infection. Additionally, this characteristic implies that the unawareness-information state r_{ui}, also extends to infinity.
